# The Effect of Donepezil Hydrochloride in the Twitcher Mouse Model of Krabbe Disease

**DOI:** 10.1007/s12035-024-04137-0

**Published:** 2024-04-01

**Authors:** Paraskevi Papakyriakopoulou, Georgia Valsami, Kumlesh K. Dev

**Affiliations:** 1 Drug Development, Department of Physiology, School of Medicine, Trinity College Dublin, Dublin 2, Ireland; 2 Laboratory of Biopharmaceutics and Pharmacokinetics, Department of Pharmacy, National and Kapodistrian University of Athens, 15784 Zografou, Greece

**Keywords:** Donepezil hydrochloride, Twitcher mouse model, Krabbe disease

## Abstract

Krabbe disease (KD) is a rare demyelinating disorder characterized by demyelination caused by mutations in the *GALC* gene, resulting in toxic accumulation of psychosine. Psychosine has been identified as detrimental to oligodendrocytes, leading to demyelination through diverse hypothesized pathways. Reducing demyelination is essential to maintain neurological function in KD; however, therapeutic interventions are currently limited. Acetylcholinesterase inhibitors (AChEi) are commonly used for symptomatic management of Alzheimer's Disease and are suggested to have potential disease-modifying effects, including regulating myelin state. In particular, donepezil, an AChEi, has demonstrated promising effects in cellular and animal models, including promotion of the expression of myelin-related genes and reduction of glial cell reactivity. This drug also acts as an agonist for sigma-1 receptors (Sig-1R), which are implicated in demyelination diseases. In the context of drug repurposing, here, we demonstrate that administration of donepezil has protective effects in the twitcher mouse model of KD. We provide data showing that donepezil preserves myelin and reduces glial cell reactivity in the brains of twitcher mice. Moreover, donepezil also improves behavioral phenotypes and increases lifespan in twitcher animals. These findings suggest that donepezil, with its dual activity as an AChE inhibitor and Sig-1R agonist, may hold promise as a therapeutic candidate for demyelinating diseases, including KD.

**Supplementary Information:**

The online version contains supplementary material available at 10.1007/s12035-024-04137-0.

## Introduction

Krabbe disease (KD) is a demyelinating disease associated with oligodendrocyte cell death and aberrant myelin state. KD, also known as globoid cell leukodystrophy, is a rare autosomal recessive disease occurring in approximately 1:100,000 [1]. This disease is a lipid storage disorder caused by mutations in the *GALC* gene, which codes for the enzyme galactosylceramidase (GALC). Mutations in *GALC* result in a loss of function and the toxic build-up of metabolites galactosylceramide and galactosylsphingosine (psychosine) [2]. Psychosine is known to be toxic to oligodendrocytes and causes demyelination by various proposed mechanisms, where myelin loss and neuroinflammation are presumably triggered by consequences of absent GALC function not clearing psychosine [3]. KD is also associated with a cluster of glial and immune cell dysfunctions including astrogliosis, microglia activation, as well as macrophage recruitment [4]. Mitigating demyelination is essential for preserving neurological function and quality of life in individuals with KD [5]. By identifying drugs that can slow or halt the progression of demyelination, the onset of the symptoms can be delayed and improved outcomes observed in individuals.

There is no approved treatment for KD, and the therapeutic approach focus on the symptomatic and supportive management of the disorder. Hematopoietic stem cell transplantation (HSCT) is the only available treatment option that has been proven to slow down the progression of KD [6]. Additionally, the Enzyme Replacement Therapy has been used successfully in other lysosomal storage disorders and is based on the intravenous administration of a synthetic form of the missing enzyme [7]. However, its efficacy in KD is limited due to the difficulty of the enzyme crossing the BBB and reaching the affected brain regions [8]. Additionally, experimental gene therapy approaches are under investigation, aiming to correct the genetic defect by introducing a functional copy of the GALC gene into the patient’s cells [9], while the supportive and symptomatic treatment focuses on managing complications, such as controlling seizures and improving the quality of life.

Acetylcholinesterase inhibitors (AChEi) impede the hydrolysis of acetylcholine (ACh), increase the levels of this neurotransmitter in the synaptic cleft and thereby enhance cholinergic transmission [10]. Cholinergic activation is considered critical for the maintenance of neuronal function and ensuring successful synaptic transmission in peripheral and central nervous systems. AChEi such as donepezil, rivastigmine, and tacrine improve cognitive function and are used for the symptomatic management of AD. Marketed AChEi drugs have been reported to have potential disease modifying targeting mechanisms. For example, these drugs appear to delay the deposition of amyloid plaques and attenuate microglia activation [11]. AChE inhibition is also related to reduce lymphocyte proliferation [12]. Cholinergic neurotransmission also plays a role in regulating the secretion of pro-inflammatory cytokines (such as TNF-α, IL-6, and IL-β). Drugs such as donepezil and rivastigmine are also suggested to play a role in regulating myelin state.

The effect of donepezil on demyelination has been assessed in cellular studies such as on oligodendrocyte precursor cells (OPCs) seeded alone [13] and in co-culture with dorsal root ganglion (OPC–DRG) neurons [14]. The drug managed to increase the length of myelinated axons and promote OPC differentiation to OLs. Moreover, the number of myelinated axons, as well as the G-ratio of remyelinated axons in the corpus callosum region of mice was found to have increased in further in vivo experiments [15]. Donepezil also upregulates the expression of myelin-related genes in OPCs [13]. AChEi drugs such as donepezil and rivastigmine are also agonists for sigma-1-receptors (Sig-1R) and bind to these receptors at a low nanomolar in range similar to AChE [15–18]. Sufficient evidence now exists to argue that efficacy of some AChEi drugs may be via a dual AChE inhibition and Sig-1R agonist mechanism. Notably, Sig-1R agonists have been suggested as potential use in demyelination diseases such as multiple sclerosis [19] and vanishing white matter (VWM) disease [20].

We have previously described a range of mechanisms that attenuate psychosine-induced cell toxicity and demyelination including (i) sphingosine 1-phosphate receptor functional antagonists [21–23], (ii) phospholipase A2 inhibitors [24], (iii) piezo 1 mechanosensitive ion channel antagonists [25], (iv) hybrid nanoparticles [26], and (v) more recently D2 receptor antagonist antipsychotics [27]. In particular, we have described that marketed drugs fingolimod [23] and haloperidol [27] promote lifespan in the twitcher mouse model of KD. In the context of drug repurposing, the myelin-promoting properties of donepezil, along with its dual inhibitory activity against AChE and activation of Sig-1R, render it as a promising candidate for the management of demyelinating diseases. Here, we aimed to assess the effect of an archetypical AChEi drug, namely donepezil, in the twitcher mouse model, a demyelinating model translatable for KD. We report that donepezil protects against the loss of myelin, dampens glial cell reactivity, slows behavioral phenotypes, and increases lifespan in the twitcher mouse model of KD.

## Materials and Methods

### Animals and Housing Conditions

Animal research adhered to EU legislation endorsed by the ethics committee of Trinity College Dublin and followed guidelines from the Health Products Regulatory Authority (HPRA) under project authorization number AE19136/P123. Genotyping of ear punch samples was conducted by TransnetYX (www.transnetyx.com). A breeding colony of heterozygous twitcher mice, acquired from Jackson Laboratory in Cambridge, UK (B6.CE-Galctwi/J Stock no: 000845), was established in a pathogen-free environment in the Comparative Medicine Unit at Trinity College Dublin using mice obtained from the Jackson Laboratory and maintained on a C57BL/6J genetic background (C57BL/6J-twi with C57BL/6J-twi). Homozygous and heterozygous animals were identified using genotyping procedure. Heterozygous animals were used only for breeding purposes and were not otherwise used in the study. The mice used in the study were housed in ventilated cages (Techniplast, IT-Varese) under specific pathogen-free conditions and constant environmental conditions (12:12 h light:dark cycle, temperature 22 ± 2 °C, relative humidity 45 ± 10%). The mice had free access to food and tap water. All mice in the facility were screened regularly according to a health-monitoring program, complying with the Federation of European Laboratory Animal Science Associations’ recommendations. The experimental protocol of the study was approved by the Health Products Regulatory Authority (HPRA, project authorization number. AE19136 /P123).

### Experimental Protocol

Donepezil hydrochloride (MW 379.50 g/mol, Cipla Ltd., India) was administered to both male and female wild-type and homozygous twitcher mice via drinking water (concentration, 20 μg/mL) [28]. Drinking water is considered as vehicle of the administered drug. Wild type and twitcher mice were used in the study, after being divided into two subgroups as follows: (a) vehicle (H_2_O) group (*n* = 8 wild type and *n* = 3 twitcher mice, for the behavioral analysis, and *n* = 5 wild type and *n* = 3 twitcher mice, for the immunohistochemistry and quantitative reverse transcription PCR) and donepezil group (*n* = 10 wild type and *n* = 7 twitcher mice, for the behavioral analysis, and *n* = 5 wild type and *n* = 5–6 twitcher mice, for the immunohistochemistry and quantitative reverse transcription PCR). The rational for using a low number of vehicle (H_2_O)-treated twicher mice (*n* = 3) was primarily based on animal ethics and we consider in line with 3R principles. In this case, we used as a benchmark, two previously published separate studies showing the effect of vehicle (H_2_O) administration in twitcher mice. Thus, the twitcher vehicle group used in this study was analyzed in comparison to previous data of twitcher mice treated with water following the same protocol (*n* = 33, Béchet et al., 2020 [23] and *n* = 8, Sharma and Dev 2023 [27]). Dose selection for oral administration was based on literature pharmacokinetic data [29], and calculated based on the daily water consumption per animal (*see supplementary material*).

### Behavioral Analysis

The behavioral analysis of wild type and twitcher mice was adapted from the previous studies [23, 27], using a modified version of the scoring systems published by Wicks et al. [30] and Beeton et al. [31] to assess the twitching frequency and mobility, respectively. Briefly, all animals were subjected to a behavioral analysis beginning at 21 days of age (postnatal day 21 (P21)), while the donepezil treatment started on the 25th day. Behavioral observations were taken daily up to three times per day, and body weight was measured at each behavior testing time point. Very mild and fine twitching, similar to post-weaning symptoms, were scored as score 1. Mild and intermittent fine twitching was given a score of 2, while constant fine twitching corresponded to a score of 3. Constant moderate twitching of body and head was given a score of 4 and was followed by euthanasia if the mouse had a score of 4, during four independent observations. Constant and severe trembling and uncontrollable twitching were given the highest score, followed immediately by euthanasia. The locomotor deficits arising due to demyelination were quantified considering that the minimum score of 1 indicates the absence of the disease, while the maximum score is 4. Fine and easy climbing with long duration was scored with 0 while late fine climbing with 1. Late and short-duration climbing had a score of 2, while the grasping and falling was scored with 3. The maximum climbing score was 4, and it was equivalent to the inability to climb. The animal should be euthanized within 24 h if moderate weakness or spasticity is noted in both hindlimbs during four independent observations. If the animal walks around and both hindlimbs remain flaccid without contributing to forward motion, it should be euthanized immediately. The genotype of the animals was performed on the 15th postnatal day (P15). The water bottles were filled by the experimenter with 250 mL of water, where the weighted amount of the drug was dissolved, and thus no blinding was included at the level of drug treatment. All data were randomized, and analysis was performed with data blinded to both genotype and drug treatment.

### HPLC Analysis

The donepezil extraction from brain tissue has been described and validated by Papakyriakopoulou et al. [32], using quercetin as internal standard (ISTD). Shortly, on the day of analysis, each brain sample was homogenized prior to analysis with a T10 ULTRA-TURRAX® (IKA Werke, DE-Staufen im Breisgau) in the presence of water for injection (WFI) (tissue:WFI ratio 1:1 w/w), and then 25 μL of homogenate tissue was vortex-mixed with 50 μL of ISTD (quercetin 2 μg/mL in methanol) and 25 μL of mobile phase. The mixture was centrifuged and 30 μL of the supernatant was received and directly injected onto the HPLC system for donepezil quantification. The high-performance liquid chromatography (HPLC) system is composed by a LC-20AD Quaternary Gradient Pump with degasser, with an SIL-HT auto-sampler and a photo-diode array detector SPD-M20A. Analysis was carried out on an analytical reverse phase MZ Analysentechnik Nucleosil 100–5 C18 column (125 × 4.6 mm, 5-μm particle size) connected to a precolumn C-18 (12.5 × 4.6 mm, 5-μm particle size, MZ Analysentechnik) of the same type. Mobile phase consisted of phosphate buffer:methanol:acetonitrile (50:40:10) and adjusted to pH 2.8 with orthophosphoric acid (80%), in isocratic mode with flow rate of 0.8 mL/min. The LC analysis time was 10 min. The injection volume was 30 μL, and the retention time of donepezil and ISTD was 4.5 and 8 min, respectively. Detection of donepezil and Que was performed at 268 and 369, respectively, and the calibration curve samples ranged from 0.05 to 3 μg/mL of donepezil.

### Processing of Tissue for IHC

Mice were euthanized by placing in a CO_2_ chamber and then transcardially perfused with 20 mL of ice-cold PBS, pH 7.4 (flow 7 mL/min), using a peristaltic pump (Watson-Marlow, Falmouth, Cornwall, UK) to remove the residual blood. Once perfusion was completed, animals were decapitated, and brains were removed and split into two hemispheres. One hemisphere was further separated into cerebellum and cortex and then stored at 80 °C until processed for quantitative reverse transcription PCR (RT-qPCR) and HPLC analysis. The remaining hemisphere was post-fixed in 4% paraformaldehyde overnight (4 °C) before being cryoprotected in 30% sucrose solution (4 °C). Brains were then snap frozen on dry ice and embedded in optimal cutting temperature compound (OCT; catalog #361603E, VWR). Parasagittal cerebellar cryosections of 12-μm thickness were cut using the Leica CM1850 Cryostat and processed for immunohistochemistry (IHC). Five animals from each treatment group were randomly selected for each primary antibody, while in the case of vehicle-treated twitchers, all the animals in the group (*n* = 3) were used for all the analyses.

### Histology and Immunofluorescence

Cerebellar cryosections were equilibrated to room temperature for 2 h and then rehydrated with 100 μL of PBS on each section. At all the steps of the procedure, the same volume was applied on every section. Sections were permeabilized using 0.2% Triton X-100 (Tx; catalog #T9284, Sigma-Aldrich) in phosphate buffer saline (PBS; catalog #18,912–014, Gibco) to reduce non-specific binding and were blocked with 10% bovine serum albumin (BSA; catalog #A3294-100G, Sigma-Aldrich) for 2 h at room temperature. Primary antibody incubations were conducted overnight at 4 °C in PBS containing 2% BSA and 0.1% Tx. Primary antibodies used were anti-myelin basic protein (MBP; 1:500 dilution; catalog #M3821-100UG, Sigma Aldrich), IbA1 polyclonal antibody (1:500 dilution; catalog #PA5-27,436, Invitrogen), and anti-vimentin (1:500 dilution; catalog #sc-373717, Santa Cruz Biotechnology). Secondary antibody incubations were performed overnight at 4 °C in PBS supplemented with 2% BSA and 0.1% Tx. The secondary antibodies used in the study were as follows: invitrogen goat anti-mouse Alexa Fluor 488 (1:500; A11001, Thermo Fisher Scientific), invitrogen goat anti-chicken Alexa Fluor 633 (1:500; A21103, Thermo Fisher Scientific), and invitrogen anti-rabbit Alexa Fluor 488 (1:500; A11008, Thermo Fisher Scientific). A counterstain with Hoechst 33,342 (1:10,000; catalog #62,249, Thermo Fisher Scientific) labeling the nucleus was performed at the end of the immunofluorescence protocol. Slices were mounted using the Invitrogen SlowFade™ Gold antifade reagent (S36936, Thermo Fisher Scientific), coverslipped on microscope slides and stored at 4 °C, in the dark, before being imaged.

### Light and Fluorescence Microscopy

Data acquisition and quantification were similar to previous studies (Sheridan and Dev, 2014). Confocal images captured for quantitative measurement of immunofluorescent staining were 12 bit.lif files of 2048 × 2048-pixel resolution and were randomly acquired throughout the cerebellum. They were captured using a Leica Sp8 scanning confocal microscope with 10 × and 20 × objective. With four cerebellar slices per slide, 10–12 images were taken per slide to cover most of the total area of the cerebellum. Five slides were used per treatment group, making a minimum of 50 images analyzed per treatment group. Image acquisition settings were kept the same across different treatments per experiment. Image analysis was conducted using the software ImageJ (https://imagej.nih.gov/ij/). Regions of interest were manually selected on each image, and fluorescence intensity was averaged. Fluorescence intensity results are shown in normalized fluorescence (% baseline) units. For Iba1 quantification, in addition to fluorescence intensity images, we used z-stacks to compute 3D models of Iba1-positive microglia with Imaris software (Bitplane). The surface of those 3D models was then used to identify the area and volume taken up by microglia, with the hypothesis that the amoeboid and reactive state of microglia in twitcher mice will be reflected in higher volume measurements.

### RT-qPCR Analysis

Total RNAs were isolated from cerebellum tissues of five randomly selected animals from each wild type group and six from donepezil-treated twitcher mice. In the case of vehicle-treated twitcher mice, all the animals in the group (*n* = 3) were used for all the analysis. The isolation was performed using the Nucleospin® total RNA isolation kit (Macherey–Nagel, Germany). The concentrations of isolated RNA from each sample were assessed using a NanoDrop 2000 Spectrophotometer (Thermo Scientific, USA). About 10 μL of the isolated RNA was equalized to 20 μL using RNase-free water. Then, the cDNA was synthesized by reverse transcription using the High-Capacity cDNA manufacture kit (Applied Biosystems™, Thermo Fisher Scientific, USA). Equal volumes of the isolated RNA and cDNA mastermix were mixed, centrifuged, and loaded in the thermal cycler (T100™, BIO-RAD, USA). The cDNA was diluted 1:2 with RNase-free water and stored at − 20 °C prior to qPCR analysis. The manufactured cDNA was amplified using multi-target qPCR. cDNA and PCR mastermix, containing the target and endogenous primers, as well as the Taqman mastermix, were loaded on a 96-well PCR fast plate (Thermo Fisher Scientific, USA) in an ice block. The plate was centrifuged (1000 rpm, 1 min) to ensure adequate mixing and then inserted into the Step One Real Time PCR System, using GAPDH (Hs02786624_g1, VIC) as endogenous control. The analysis was performed on specific gene-targets, using myelin markers (MBP, myelin oligodendrocyte glycoprotein: MOG), astrocyte (*VIMENTIN*) and microglia (*IBA1*) markers, as well as the proinflammatory (*IL-6*) gene. Gene expression data were analyzed using the Livak and Schmittgen (2001) [33] ΔΔC_t_ method, to produce a relative quantity (*RQ*) value of target gene expression relative to GAPDH expression as per formula in Eqs. (1)–(3): (1) ΔC_t_ = C_t_ target − Ct endogenous; (2) ΔΔC_t_ = ΔC_t_ sample − ΔC_t_ control sample; (3) *RQ* = 2^−ΔΔCt^.

### Inflammatory Gene and Protein Expression Signatures

Inflammatory gene and protein expression signatures were generated for each animal using the 5 genes (*MBP, MOG, VIMENTIN, IBA-1*, and *IL-6*). For the genes/proteins that are positively associated with myelin (e.g. *MBP* and *MOG*), the original data were used. Conversely, for the genes/proteins negatively associated with myelin (e.g., glia reactivity and inflammation), inverse data were used. Subsequently, the inverted data of *VIMENTIN*, *IBA-1*, and *IL-6* genes/proteins were averaged and divided by the average value of *MBP* and *MOG* genes/proteins’ original data to produce the inflammation/myelin ratio. This ratio serves as an optimal signature method for the gene and protein expression of each individual animal.

### Statistical Analysis

Experimental data were analyzed and graphically represented using GraphPad Prism 8.0 software package (GraphPad Software). Data normality was assessed using the Shapiro–Wilk test. The difference in the life span between twitcher and wild-type mice was tested applying the Kaplan–Meier log-rank analysis. Discrepancies in weight gain, twitching severity, and mobility impairments were analyzed using the two-way ANOVA with Bonferroni post hoc test for multiple comparisons. The minimum level of significance was set at *p* < 0.05. Mean fluorescence intensity was measured with ImageJ software and used as an arbitrary unit of measure. Raw datasets were normalized and presented as percentages of the control group (vehicle-treated wild type animals) average. For that purpose, each arbitrary value in the experimental group was divided by the mean of the control group and multiplied by 100. The possible differences among the animal groups during the IHC and RT-qPCR analysis were assessed applying the one-way ANOVA with Bonferroni post hoc test for multiple comparisons, as well as Mann–Whitney non-parametric test between all group pairs. Graphical data are represented as the mean ± SEM.

## Results

### Donepezil Enhances the Levels of Myelin in Cerebellum of Twitcher Mice

**Fig. 1:**
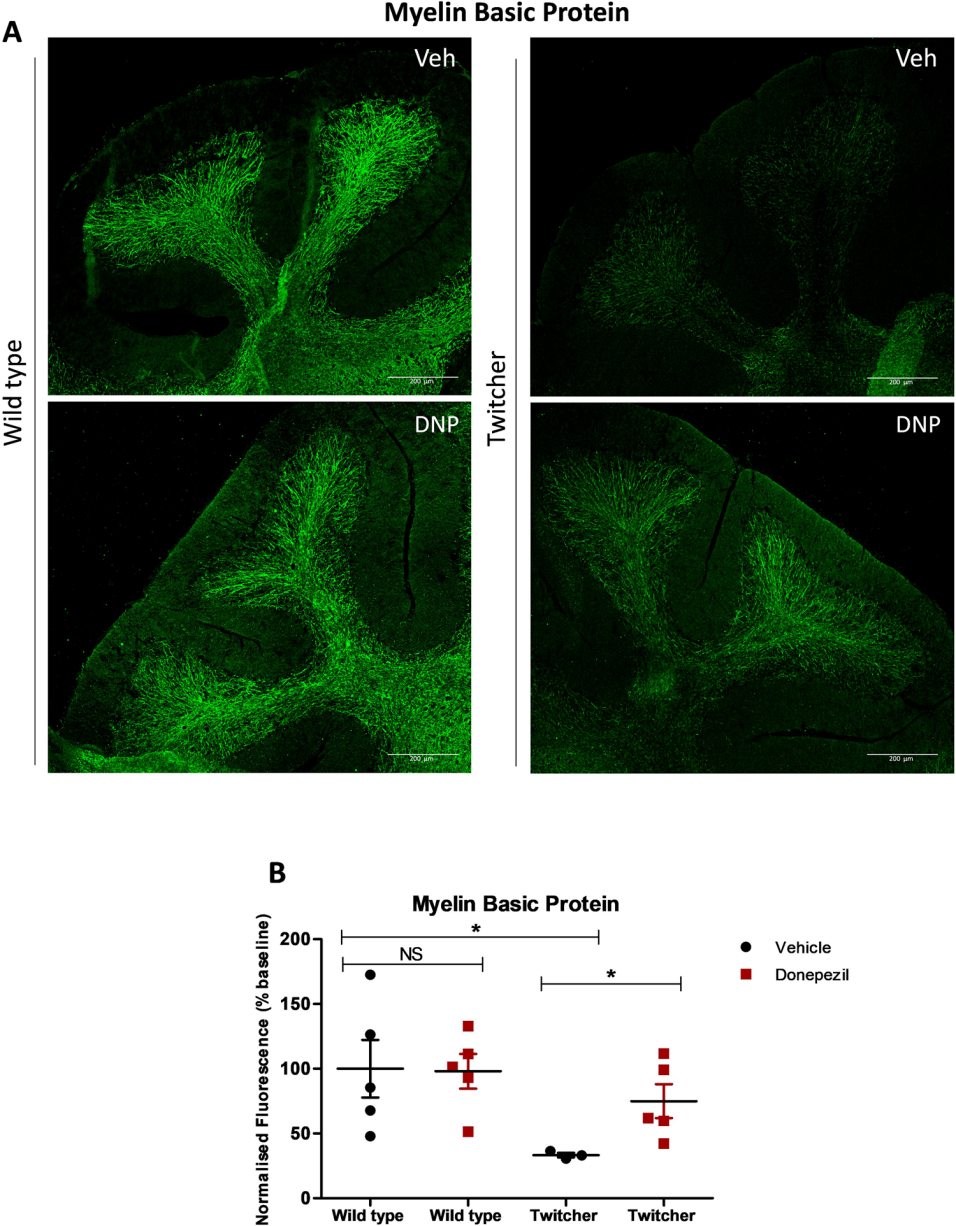
Donepezil increases levels of MBP in twitcher mice. **A** Representative confocal images display MBP immunostaining under the different treatment conditions (vehicle, Veh; donepezil, DNP). The images were captured at 10 × magnification, focusing on cerebellar lobes. Scale bar, 200 μm. **B** Graphs illustrating the donepezil-treatment effect on MBP normalized fluorescence (% baseline). Differences between the treatment groups were statistically analyzed applying one-way ANOVA followed by a Bonferroni correction for multiple comparisons, as well as Mann–Whitney non-parametric test between all group pairs (NS, not significant, * *p* < 0.05, *n* = 3–5 animals). Graphical data are represented as the *mean* ± *SEM*

The effects of donepezil in myelin restoration have been reported in OPC–DRG neuron co-culture, as well as in a cuprizone-induced demyelination mouse model [12]. Therefore, we examined the effects of this drug on levels of MBP in twitcher mice. The treatment of wild type animals with donepezil did not alter basal levels of MBP (100 ± 28.7 vehicle vs 98 ± 13.4% donepezil, *p* = 0.841, *n* = 5) (Fig. 1), in agreement with previous studies [34]. As expected, the levels of MBP were significantly reduced in vehicle-treated twitcher mice compared to wild type littermates (100 ± 28.7 wild type vs 33 ± 1.7% twitcher, * *p* = 0.036, *n* = 3–5) (Fig. 1). Notably, administration of twitcher mice with donepezil significantly improved MBP expression, compared to untreated animals, where the levels of MBP in donepeziltreated twitcher mice were found approaching those of wild type animals (33 ± 1.7 vehicle vs 75 ± 13.1% donepezil, * *p* = 0.036, *n* = 3–5) (Fig. 1).

### Donepezil Alters Vimentin Expression in White Matter Layer of Cerebellum in Twitcher Mice

**Fig. 2:**
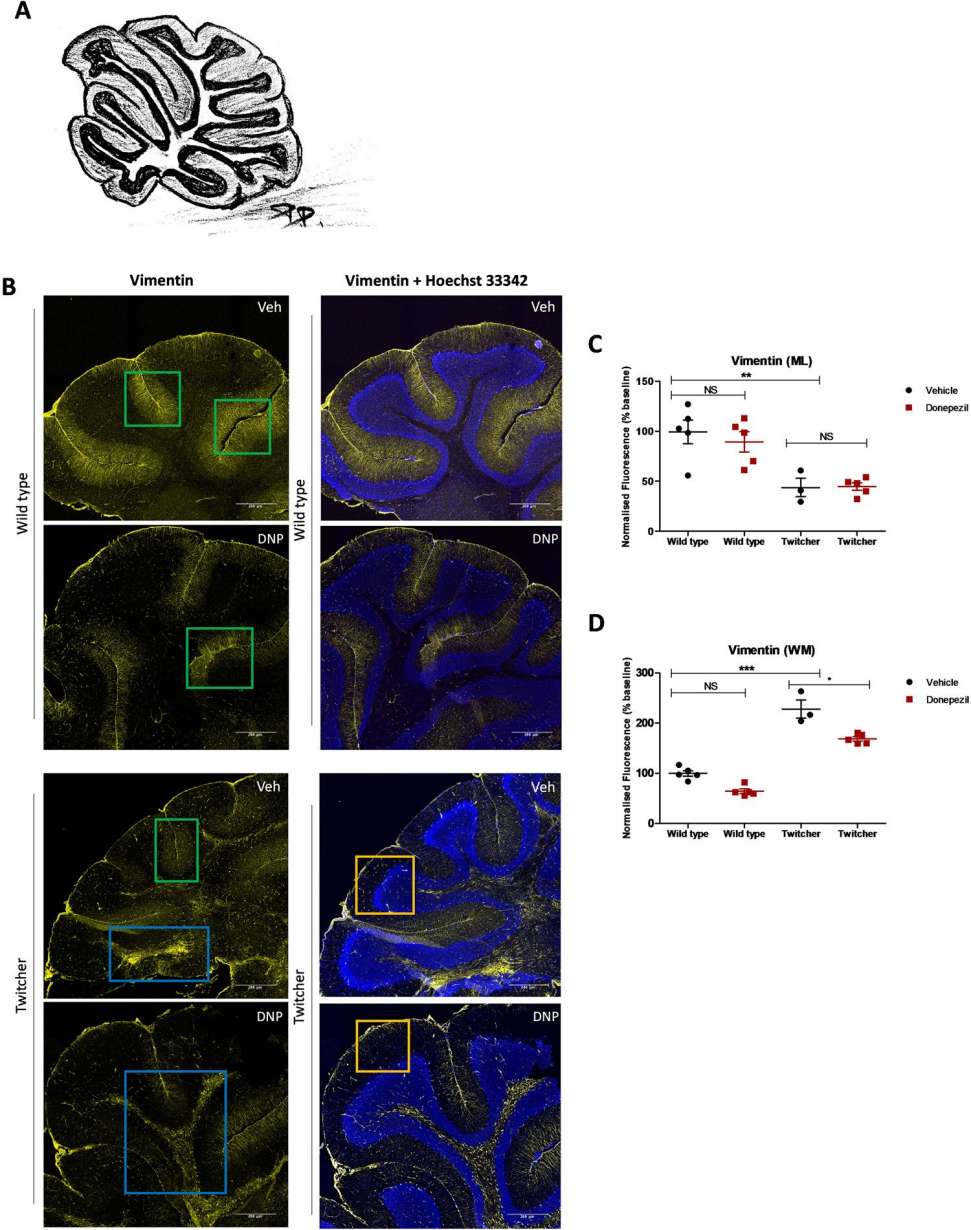
Effects of donepezil on astrocytes in twitcher mice. **A** Sagittal mouse cerebellum scheme illustrating the different anatomical layers; molecular layer (ML), granular layer (GL), and white matter (WM). **B** Representative confocal images display Vimentin immunostaining, alone and with Hoechst 33,342 counterstain under the different treatment conditions (vehicle, Veh; donepezil, DNP). The images were captured at 10 × magnification, focusing on cerebellar lobes. Scale bar, 200 μm. Graphs illustrating the donepezil-treatment effect on Vimentin normalized fluorescence (% baseline) at the **C** Bergmann glial cells of molecular layer (ML), and **D** fibrous astrocytes of white matter (WM). Differences between the treatment groups were statistically analyzed applying one-way ANOVA followed by a Bonferroni correction for multiple comparisons, as well as Mann–Whitney non-parametric test between all group pairs (NS, not significant, * *p* < 0.05, ** *p* < 0.01, *** *p* < 0.001, *n* = 3–5 animals). Graphical data are represented as the *mean* ± *SEM*. The areas of interest in ML, GL, and WM, are noted with green, orange, and blue squares, respectively

KD progression is accompanied by astrocyte reactivation followed by the same event in microglia [20]. Astrocytes in the layers of the cerebellum include the Bergmann glial cells of molecular layer (ML) and granular layer (GL) and fibrous astrocytes of white matter (WM) (Fig. 2A). The type III intermediate filament astrocyte marker vimentin was employed to assess the effects of donepezil on astrocytes in twitcher mice. In agreement with our previous studies [23], the fluorescence levels of vimentin in ML were decreased significantly in twitcher mice compared to wild type littermates (Fig. 2B, green squares) (100 ± 14.6 vs 48 ± 10.1, ** *p* = 0.0029, *n* = 3–5) (Fig. 2C). These levels of vimentin in the ML were not altered by administration of donepezil either in wild type animals (100 ± 14.6 vehicle vs 89 ± 10.1 donepezil, *p* = 0.7533, *n* = 3–5) or twitcher animals (48 ± 10.1 vehicle vs 50 ± 4.3 donepezil, p = 1.000, n = 3–5) (Fig. 2B,C). In contrast, in twitcher mice treated with vehicle, we noted a translocation of vimentin fluorescence from the ML to GL (Fig. 2B, orange squares) and the formation of fibrous astrocytes aggregations in the WM (Fig. 2B, blue squares) (100 ± 7.0 wild type vs 228 ± 18.1 twitcher, *** *p* < 0.0001, *n* = 3–5) (Figure D), which was partially reversed by treatment with donepezil (228 ± 18.1 vehicle vs 169 ± 4.4 donepezil, * *p* = 0.0357, *n* = 3–5) (Fig. 2B,D).

### Donepezil Decreases Iba1 Expression in the Cerebellum of Twitcher Mice

**Fig. 3:**
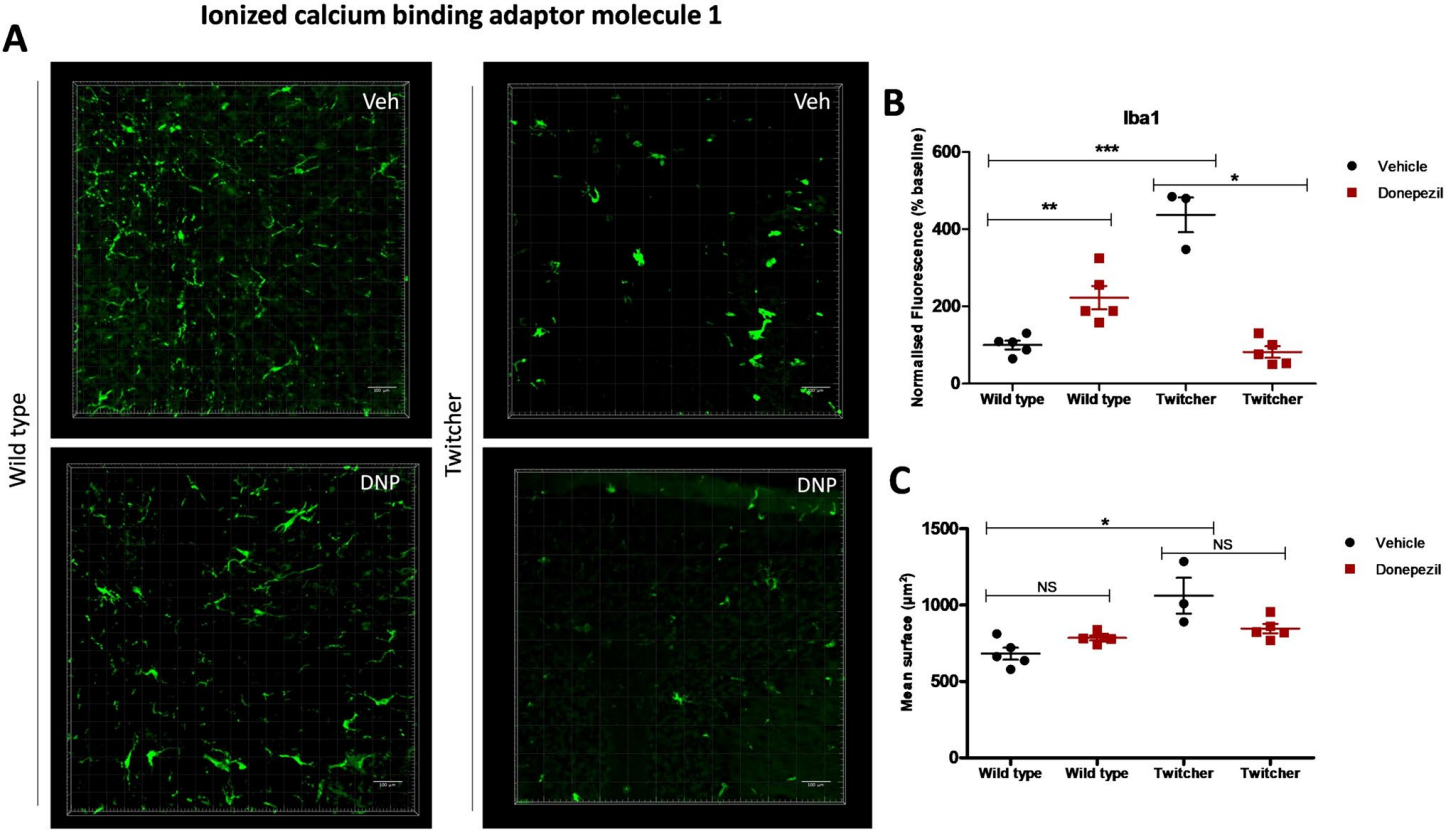
Evaluation of microglia in wild type and twitcher mice treated with donepezil.** A** Representative images created using IMARIS software display Iba1 immunostaining under the different treatment conditions (vehicle, Veh; donepezil, DNP). The images were captured at 20 × magnification, focusing on cerebellar lobes. Scale bar, 100 μm. **B** Graph illustrating the donepezil-treatment effect on Iba1 normalized fluorescence (% baseline). **C** Graph illustrating the Iba1-positive areas expressed in square micrometers. Differences between the treatment groups were statistically analyzed applying one-wa–-Whitney non-parametric test between all group pairs (NS, not significant, * *p* < 0.05, ** *p* < 0.01, *** *p* < 0.001, *n* = 3–5 animals). Graphical data are represented as the *mean* ± *SEM*

The role of microglia in KD development and progression has been characterized as critical for globoid cell formation in twitcher mouse brain [35]. Microglia activation occurs in early-stage KD and can induce astrogliosis, as well as promote myelin loss and neurodegeneration [36]. Donepezil inhibitory effect against microglia activation has been reported in microglia/neuroblastoma coculture and C57BL/6J mice given 10 mg/kg intraperitoneally and was characterized as independent of ACh receptor signaling [37]. In this study, the fluorescence data for ionized calcium binding adaptor molecule 1 (Iba1) marker of microglia/macrophage revealed a significant effect of donepezil in wild type and twitcher-treated animals (Fig. 3). Namely, the drug reduced the expression of Iba1 in the cerebellum of twitcher mice (437 ± 44.8 vehicle vs 82 ± 15.2% donepezil, * *p* = 0.036, *n* = 3–5) (Fig. 3A,B), while increasing the respective levels in wild type littermates (100 ± 14.4 vehicle vs 223 ± 30.0% donepezil, ** *p* = 0.0079, *n* = 3–5) (Fig. 3A,B). Analysis of mean surface area of Iba1 immunofluorescence showed a significant increase between vehicle-treated wild-type and twitcher animals (682 ± 39.6 wild-type vs 1061 ± 117 twitcher, * *p* = 0.0357) (Fig. 3A,C). Treatment with donepezil diminished these differences, where the mean surface data did not show significant variation between the vehicle and donepezil-treated animals, of both wild type (682 ± 39.6 vehicle vs 786 ± 18.1 donepezil, *p* = 0.0952, *n* = 3–5) and twitcher groups (1061 ± 117 vehicle vs 846 ± 31.2 donepezil, *p* = 0.0714, *n* = 3–5) (Fig. 3A,C). The immunostaining revealed the amoeboid morphology in twitcher mice cerebellum, compared to the ramified microglia of wild type animals, as well as the higher Iba1 intensity ascribed to the inflammatory state of KD (Fig. 3A).

### Donepezil Effect on a Combined Myelin, Glial, Inflammatory Gene Expression Signature

**Fig. 4:**
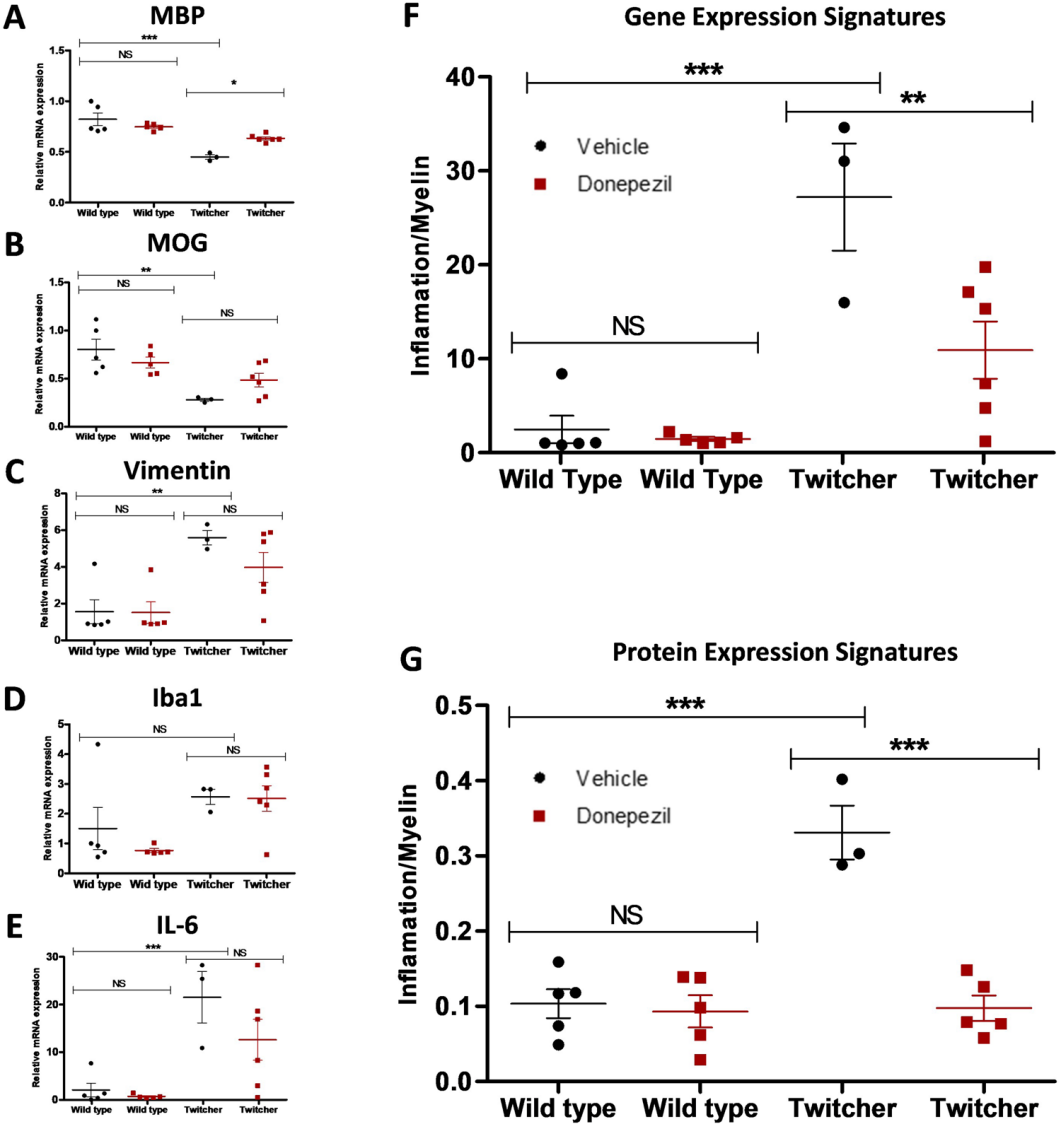
Evaluation of donepezil effect on a combined myelin, glial, inflammatory gene expression signature. Graphs illustrating the donepezil-treatment effect on relative mRNA expression of **A** *MBP*, **B** *MOG*, **C** *VIMENTIN*, **D** *IBA1*, and **E** *IL-6* genes using RT-qPCR analysis. **F** Graph shows algorithm analysis of gene-expression-signature using RT-qPCR data in which an average of expression values for glia and cytokine inflammatory markers (*VIMENTIN*, *IBA1*, and *IL-6*) was divided by an average of expression values for myelin markers (*MBP* and *MOG*), for each individual animal. **G** Graph shows algorithm analysis of protein-expression-signature using immunocytochemistry data in which an average of expression values for glia cell markers (*VIMENTIN* and *IBA1*) was divided by an average of expression values for myelin markers (*MBP* and *MOG*), for each individual animal. Differences between the treatment groups were statistically analyzed applying one-way ANOVA followed by a Bonferroni correction for multiple comparisons, as well as Mann–Whitney non-parametric test between all group pairs (NS, not significant, * *p* < 0.05, ** *p* < 0.01, *** *p* < 0.001, *n* = 3–5 animals). Graphical data are represented as the *mean* ± *SEM*

KD is associated with alterations in myelin-, glial-, and inflammatory-markers. In order to create a biomarker-signature of this disease model, we initially examined the effects of donepezil on individual gene expression of the myelin markers (*MBP* and *MOG*) (Fig. 4A,B), as well as on glia cells astrocyte (*VIMENTIN*) (Fig. 4C) and microglia (*IBA1*) (Fig. 4D) markers, and a pro-inflammatory cytokine (*IL-6*) (Fig. 4E), using RT-qPCR analysis. The RT-qPCR analysis demonstrated the lower gene expression of MBP and MOG in the cerebellum of twitcher mice, while showing higher levels of *VIMENTIN*, *IBA1* and *IL-6*, suggestive of a loss of myelin, enhanced glial cell reactivity and pro-inflammatory phenotypes (Fig. 4A-E). Next, we created an algorithm to generate a gene-expression-signature in which an average of expression values for glia and cytokine inflammatory markers (*VIMENTIN*, *IBA1*, and *IL-6*) was divided by an average of expression values for myelin markers (*MBP* and *MOG*), for each individual animal (Fig. 4F). These signatures showed no significant difference (*p* = 0.2495) in wild-type mice treated with vehicle or donepezil, as expected. Importantly, an enhanced ratio of glial cell and pro-inflammatory markers versus myelin markers was observed in twitcher mice compared to wild-type mice (*p* = 0.0001). Moreover, the administration of twitcher mice with donepezil showed a significant decrease (*p* = 0.0016) in this gene expression signature compared to vehicle-treated twitcher mice (Fig. 4F). A similar algorithm was also generated for immunocytochemistry analysis, where an average of expression values for glia cell markers (*VIMENTIN* and *IBA1*) was divided by an average of expression values for myelin markers (*MBP* and *MOG*), for each individual animal (Fig. 4G). These protein expression signatures agreed with gene expression signature data, showing enhanced glia cell reactivity versus myelin levels in twitcher mice (*p* < 0.0001) that was significantly attenuated in twitcher mice treated with donepezil (*p* < 0.0001) (Fig. 4G).

### Donepezil Increases the Lifespan of Twitcher Mice

**Fig. 5:**
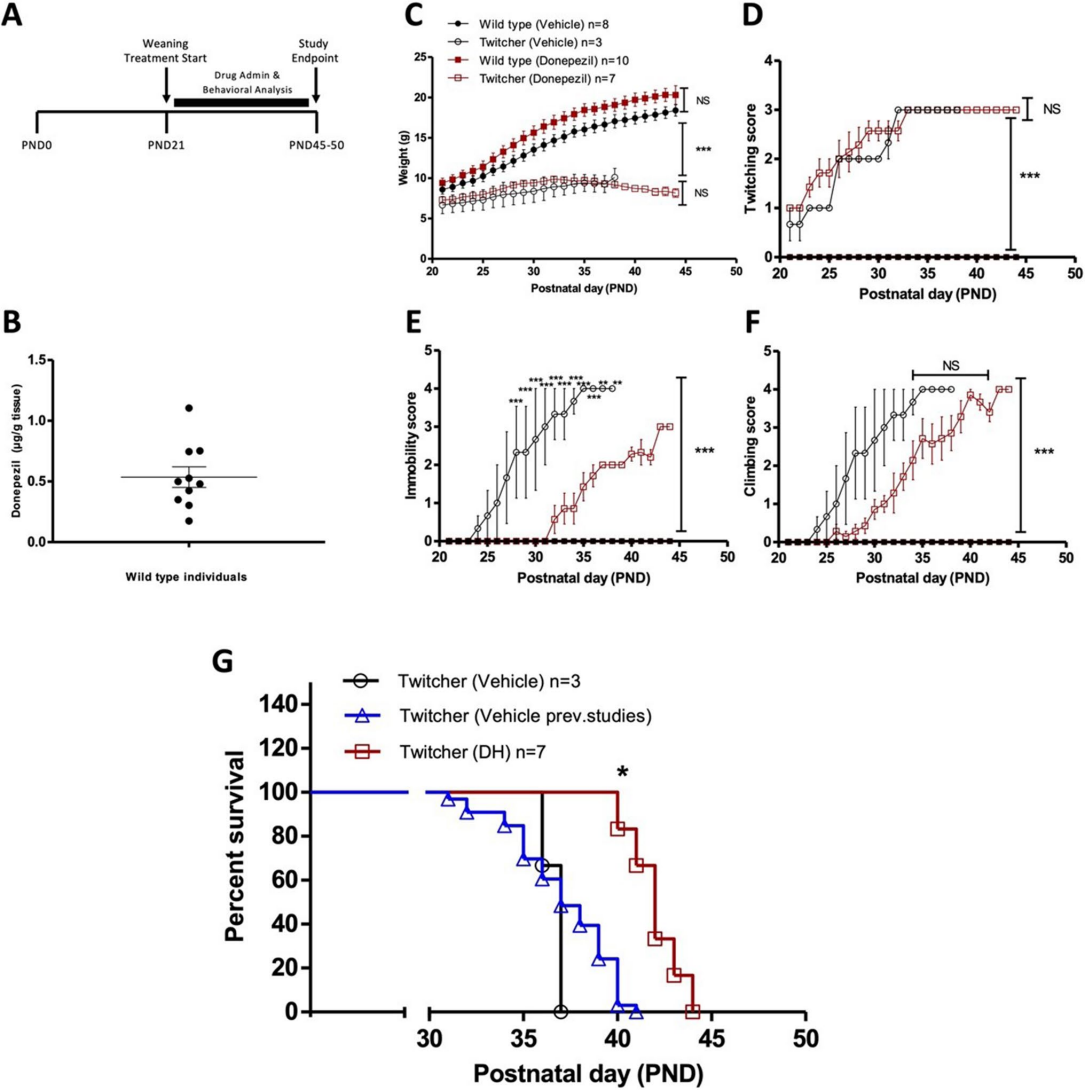
Donepezil enhances the lifespan of twitcher mice.** A** Schematic representation of the study design. Donepezil treatment commenced at P21 with daily behavior monitoring. **B** Brain concentration levels of donepezil in wild type animals, after 44 days of daily administration via the drinking water (*n* = 10). **C** Body weight of wild type and twitcher mice treated with vehicle or donepezil (two-way ANOVA followed by a Bonferroni adjustment for multiple comparisons, (*** *p* < 0.001, *n* = 3–8 animals). Behavioral analysis of donepezil-treatment effect on **D** twitching, **E** immobility, and **F** climbing score (two-way ANOVA followed by a Bonferroni adjustment for multiple comparisons (NS, not significant, ** *p* < 0.01, *** *p* < 0.001, *n* = 3–8 animals). **G** The Kaplan–Meier survival curve depicts the lifespan increase of donepezil-treated versus untreated twitcher mice. The survival curve (blue line) of untreated twitcher mice from previous studies [23, 27] were included in the graph for comparison. A log-rank Mantel–Cox test reveals that the donepezil-treated twitcher group (*n* = 7) was significantly different from vehicle-treated groups (current study, *n* = 3, * *p* < 0.05; previous studies, *n* = 33, * *p* < 0.05)

Administration of drug (donepezil) and behavioral analysis was initiated upon weaning of animals at PND21. The immunocytochemistry and RT-qPCR analysis indicated in the data above was conducted at the study endpoint (Fig. 5A). Drug brain levels of donepezil were measured in wild type littermates treated with donepezil via drinking water for 44 days using HPLC analysis (Fig. 5B). The concentration values correspond to the brain drug levels on the day of sacrifice (P44), and they are normally distributed around the mean value (*p* = 0.6753). Outlier detection occurred by applying the interquartile range (*IQR*) using a step of 1.5 × *IQR*. No outliers were detected (*p* > 0.05, for all the tested individuals). We note the concentration of donepezil in the brains of twitcher mice were not detectable when sacrificed. This finding is in line with the daily water consumption of mice, which decreases from 32 PND to the end-stage of life, affecting the DNP daily dose per animal (see supplementary material). From 32 PND onwards, the animals’ hydration mostly occurred via a drug-free gel food.

Donepezil oral administration in twitcher mice via the drinking water from P25 onward showed an increased trend in the body weight from the P27 to P32 compared to vehicle-treated twitcher animals, which was not different between these two twitcher groups from P32 onwards to end stage of life (Fig. 5C). Twitching scoring was progressive with disease in twitcher mice and did not reveal any significant difference between the donepezil-treated and vehicle-treated animals (Fig. 5D), where both groups plateaued at P32–P33, until the endpoint of the study. In the case of immobility score, a significant positive effect of donepezil treatment is noted from the P28 onwards, where the drug delayed appearance of immobility by approximately 8 days as well as decreased severity of this phenotype (Fig. 5E). We also monitored the natural climbing ability of mice on the cage rack and noted that twitcher mice showed decreased climbing score compared to wild-type littermates (Fig. 5F). In this case, we scored fine and easy climbing with long duration at 0 (as seen in wildtype mice and twitcher mice until 20–25 days of age), while late fine climbing was scored with 1, late and short-duration climbing had a score of 2, grasping and falling was scored with 3, and the inability of climbing was scored at 4. The loss of climbing behavior was delayed in donepezil-treated twitcher mice compared to vehicle-treated (Fig. 5F). These overall mobility assessments constitute an efficacy of donepezil in the management of behavioral symptoms in twitcher mice.

The effect of donepezil on lifespan of twitcher animals was also examined (Fig. 5G). In the current study, we note a caveat in our decision to use of a small group size for vehicle-treated twitcher animals (*n* = 3). We took this option due to (i) primarily, ethical considerations to limit use of the severe model of twitcher; (ii) also, given we have a recent body of evidence from our previous observations that no vehicle-treated twitcher animal typically survives beyond 30–40 days (*n* = 33) [23, 27], and (iii) because, we are seeking therapies which promote lifespan of twitcher to beyond 40 days or more. Our current findings treating twitcher mice with vehicle show as lifespan of 36 days (*n* = 3) (Fig. 5G), in agreement with our previous observations (*n* = 33) [23, 27]. Importantly, the Kaplan–Meier survival curves demonstrate that donepezil treatment leads to a median lifespan of 42 days in twitcher mice (ranging from 40 to 44), while the 43% of them reached the 43rd day, which was statistically increased (*p* = 0.0108) compared to vehicle-treated twitcher mice in our current study and previous data (Fig. 5G).

## Discussion

### Summary of findings

Krabbe disease (KD) is associated with genetic mutations and subsequent loss of function of the acid hydrolase galactosylceramidase (GalC) enzyme, which plays a key role in metabolism of galactosylceramide and the toxic galactolipid galactosylsphingosine (psychosine) [38]. Strong evidence has been established about the contribution of psychosine accumulation in nervous system and its toxic effects on levels of myelin [39]. Psychosine accumulation has been determined throughout the CNS, in the spinal cord, cerebrum, and cerebellum [40, 41]. A natural occurring animal model of KD, namely the twitcher mouse, displays myelin damage accompanied by axonal degeneration and the formation of globoid cells in the white matter and is well recognized as translational model of the disease [42]. With studies reporting positive effects of AChE inhibitors and Sig-1R agonists on levels of myelin, that donepezil regulates levels of myelin and oligodendrocyte function, and that this marketed drug has a dual activity as an AChE inhibitor and Sig-1R agonist, we hypothesized that donepezil would display efficacy in the demyelinating twitcher mouse model of KD. The present results provide a direct demonstration that donepezil rescues deficits seen at the pathological level in twitcher’s mice, namely that donepezil oral treatment led to significant increase of myelin levels, restoration of astrocyte reactivity, as well as suppression of microglia activation. The current data also demonstrates that donepezil enhances mobility of twitcher animals as depicted by immobility and climbing scores, as well as promote life span in these animals.

### The Dual Role of AChE and Sig-1R Receptors in Myelination

Myelin regeneration requires the gradual replacement and renewal of OLs through a two-stage process including the differentiation of OPCs into OLs and the production of myelin sheaths around the neuronal axons. This axonal wrapping is necessary for the transmission of electrical signals along the axon [43]. The muscarinic acetylcholine receptors (mAChRs) M1, M3, and M4 have been identified in OPCs, while all the mAChR subtypes are reported in low levels in mature OLs [44]. Furthermore, the co-expression of GalC and ACh transporter in a large portion of OLs population, as well as the decrease of GalC in the presence of a cholinergic antagonist, suggests the involvement of ACh receptors in the differentiation of OPCs [45]. However, only donepezil, among other AChEi such as tacrine, physostigmine, and galantamine, has been reported to display myelin-promoting effects in OPC cell culture [11].

Sig-1Rs have been reported to play a role in regulating levels of myelin [16–18]. Apart from its ability to inhibit the AChE displacing the active site of enzyme [46], donepezil is an agonist of Sig-1R with affinity and efficacy at low nanomolar concentrations [13]. Positron emission tomography (PET) scan studies in both humans and rodents have also demonstrated that donepezil can bind to these receptors at therapeutic doses (5 and 10 mg produce 60 and 75% Sig-1R occupancy in humans) [14, 15]. The donepezil EC50 value that ensures the receptor occupancy in rats was found to be 333.0 nM, corresponding to 1.07 mg/kg [15]. Hence, here, we propose a dual interaction with both AChE inhibition Sig-1R activation may be responsible for donepezil remyelinating mechanism. Several Sig-1R agonists have been developed; some of which are in clinical studies and others that are market approved; however, none has been tested in an animal model of Krabbe disease.

### Donepezil Effects on Astrocytes and Microglia Reactivity and Pro-inflammatory Phenotype

It is well-established that astrocytes and microglia play as critical role in myelin generation as regulators of migration, proliferation, and maturation stages towards the production of myelinating OLs [47]. Both nicotinic (nAChRs) and muscarinic (mAChRs) receptors are also known to be expressed by astrocytes and microglia and response to changes in levels of ACh neurotransmitter [48–50]. Thus, it is possible that enhanced levels of ACh in the brains of twitcher mice treated with donepezil may constitute a mechanism by which this drug induces the anti-inflammatory effects we observed on these glia cells.

Donepezil is also a well-known agonist of Sig-1Rs, where these receptors are involved in the modulation of astrocytes and microglia and regulate permeability of the blood–brain-barrier [51], perhaps providing another avenue for this drug to elicit its effects in twitcher mice. The increased levels of Iba1 expression in wild type mice can be attributed to Sig-1R and a7nAChR both of which are expressed in microglial cells [52, 53]. As mentioned above, Sig-1R activation contributes to the inhibition of AChE, as well as to calcium mobilization through IP3-gated ion channels and voltage-gated potassium and calcium channels, thereby increasing the levels of ACh [54]. The enhancement of acetylcholine levels by donepezil treatment could potentially lead to increased microglial activation. However, this activation is not considered pathological, as the ramified morphology of microglia is not affected, in contrast to the amoeboid morphology noted in twicher mice. Additionally, the high expression of Sig-1R in astrocytes can explain the positive effect of donepezil treatment in WM (Fig. 2B, green squares). This effect can be related to the promotion of oligodendrogenesis and/or the functional recovery of WM [55], albeit moderately expressed, without allowing for the complete restoration of Bergmann cells’ position, back to ML (Fig. 2A,B, orange squares).

Donepezil is also known to attenuate Aβ-induced gliosis in microglia cells and microglia activation in an AD mouse model [56]. Pro-inflammatory signals such as IL-6 have been reported importantly enhanced in the pathological condition of KD and twitcher mice [57], and in agreement with our findings, donepezil can lower neuroinflammation after the treatment [58]. Our data reinforces the argument of donepezil anti-inflammatory properties and supports previous literature [59].

### Challenges in Enhancing the Lifespan of Twitcher Animals

Our observations of donepezil effects on myelin state, astrocyte and microglia reactivity, and pro-inflammatory phenotypes are reflected in twitcher mouse phenotype as a 5-day life extension and improvement of mobility characteristics. The benefit of donepezil oral administration in this animal model is commensurate with the effectiveness of other marketed drugs such as fingolimod or haloperidol assessed in our previous studies [23, 27]. The effects of these drugs are likely explained by multiorgan impairment, observed in previous works on twitcher mouse model [60, 61], which unless restored, likely supersedes attempts to solely rescue dysfunction in the nervous system. The body-wide damage caused by the buildup of psychosine thereby limits the efficacy of drugs such as fingolimod, haloperidol, and donepezil to show lasting improvement in twitching and mobility or increase the lifespan further, as previously suggested [23, 27]. Nevertheless, the overall ameliorated phenotype and achieved increase of lifespan after donepezil treatment may not be considered negligible given the severity of this KD translational model. In conclusion, we find that donepezil has myelin-promoting properties, as well as activity against neuroinflammation and show this drug prolongs lifespan of twitcher mice. The findings constitute encouraging results that allow the conceptualization of dual targeting of AChEi and Sig-1R in management of demyelinating diseases, such as Krabbe disease.

## Supplementary Information

Below is the link to the electronic supplementary material.

Supplementary file1 (PDF 308 KB)

## Data Availability

Data will be made available on reasonable request.
