# Supplementary material for: The Effect of Donepezil Hydrochloride in the Twitcher Mouse Model of Krabbe Disease
Source: Mol Neurobiol. 2024 Apr 1;61(11):8688–701. doi: 10.1007/s12035-024-04137-0 (PMC11496341; doi:10.1007/s12035-024-04137-0)
Supplement: Supplementary file 1 — Supplementary file1 (PDF 308 KB) [file 12035_2024_4137_MOESM1_ESM.pdf]

Supplementary material

Title: The effect of donepezil hydrochloride in the Twitcher mouse model of Krabbe's disease

Paraskevi Papakyriakopoulou[1,2], Georgia Valsami[2,*] and Kumlesh K. Dev[1,*]

[1] Drug Development, Department of Physiology, School of Medicine, Trinity College Dublin, Ireland

[2] Laboratory of Biopharmaceutics and Pharmacokinetics, Department of Pharmacy, National and Kapodistrian University of Athens, 15784, Greece

***Correspondence should be addressed to:**

Prof. Kumlesh K. Dev, devk@tcd.ie

Prof. Georgia Valsami, valsami@pharm.uoa.gr

1. Dose calculation based on water consumption

The daily water consumption per cage was measured during the experiment and defined for wild type and twitcher mice, respectively. Considering the volume decrease in the bottle and the number of animals in each cage, the mean daily water consumption (mL) per animal, either for wild type or twitcher, was calculated and presented in Table S1 and Figure S1. Additionally, the daily water consumption (mL) per body weight (g) was also calculated and included in Table S1. The daily DNP dose for each animal was calculated based on the addition of 5 mg in 250 mL and considering the water volume (mL) for each animal, while the daily DNP dose (mg) per body weight (g) was also calculated and included in Table S2 and Figure S2.

Based on the trends of Figures S1,2 it can be assumed that a significant reduction in water consumption of twitcher mice takes place at 32nd PND onwards, until the complete cessation of DNP uptake from 38 PND to the end-stage of life. Accordingly, the administered dose was found to be decreased from 32nd PND onwards due to the progression of the disease, which renders it more difficult for the animal to access the water bottle.

Table S1. Mean daily water consumption (mL) per animal and mean daily water consumption (mL)/body weight (g), for wild type and twitcher mice.

Day →			**25**	**26**	**27**	**28**	**29**	**30**	**31**	**32**	**33**	**34**	**35**	**36**	**37**	**38**	**39**	**40**	**41**	**42**	**43**
**Water volume (mL) per animal**	**WT**	MEAN	3.3	3.5	3.7	4.0	4.2	4.3	4.5	4.7	4.8	5.0	5.2	5.2	5.3	5.5	5.3	5.5	5.3	5.5	5.5
	**TWI**	MEAN	0.83	1.75	2.67	2.50	2.92	3.33	3.25	0.17	0.58	0.50	0.17	0.42	0.08	**0.00**	**0.00**	**0.00**	**0.00**	**0.00**	**0.00**
**Water (mL)/ body weight (g)**	**WT**	MEAN	0.26	0.26	0.26	0.27	0.27	0.26	0.26	0.26	0.26	0.27	0.27	0.26	0.27	0.27	0.26	0.26	0.25	0.25	0.25
		SD	0.01	0.01	0.01	0.01	0.01	0.01	0.01	0.01	0.00	0.00	0.01	0.01	0.01	0.01	0.01	0.01	0.01	0.01	0.01
	**TWI**	MEAN	0.107	0.209	0.303	0.270	0.306	0.354	0.335	0.016	0.058	0.051	0.017	0.044	0.009	**0.000**	**0.000**	**0.000**	**0.000**	**0.000**	**0.000**
		SD	0.007	0.018	0.023	0.017	0.024	0.031	0.029	0.001	0.005	0.005	0.002	0.005	0.001	0.000	0.000	0.000	0.000	0.000	0.000

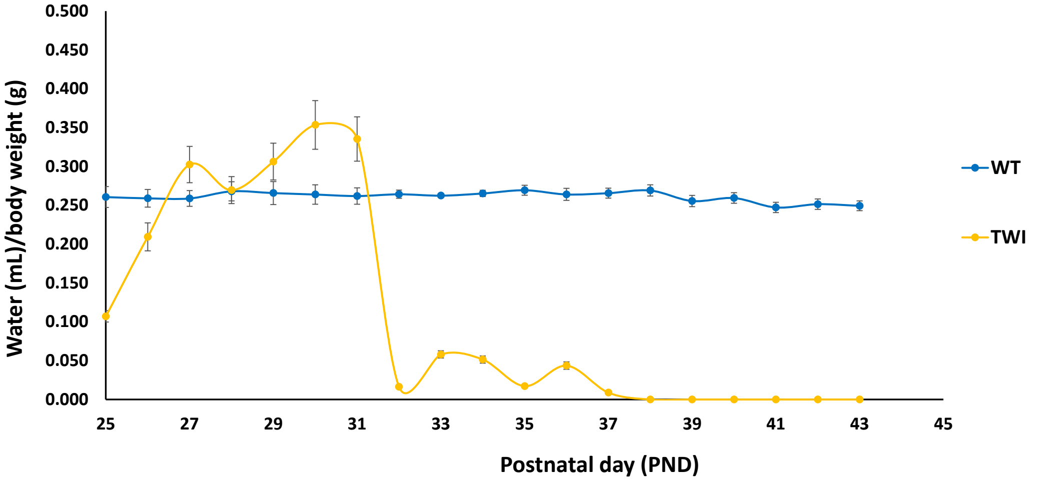

Figure 1. Mean daily water consumption (mL) per animal and (± SD), mean daily water consumption (mL)/body weight (g)for wild type (blue line) and twitcher mice (yellow line).

Table S2. Mean daily DNP dose (mg)/per animal either for wild type and twitcher mice

Day →			25	26	27	28	29	30	31	32	33	34	35	36	37	38	39	40	41	42	43
DNP (mg)/ per animal	WT	MEAN	0.005	0.005	0.005	0.005	0.005	0.005	0.005	0.005	0.005	0.005	0.005	0.005	0.005	0.005	0.005	0.005	0.005	0.005	0.005
	TWI	MEAN	0.002	0.004	0.006	0.005	0.006	0.007	0.007	0.000	0.001	0.001	0.0003	0.001	0.0002	0.000	0.000	0.000	0.000	0.000	0.000
DNP (mg)/ mean body weight (g)	WT	MEAN	0.005	0.005	0.005	0.005	0.005	0.005	0.005	0.005	0.005	0.005	0.005	0.005	0.005	0.005	0.005	0.005	0.005	0.005	0.005
		SD	0.000	0.000	0.000	0.000	0.000	0.000	0.000	0.000	0.000	0.000	0.000	0.000	0.000	0.000	0.000	0.000	0.000	0.000	0.000
	TWI	MEAN	0.002	0.004	0.006	0.005	0.006	0.007	0.007	0.000	0.001	0.001	0.000	0.001	**0.000**	**0.000**	**0.000**	**0.000**	**0.000**	**0.000**	**0.000**
		SD	0.000	0.000	0.000	0.000	0.000	0.001	0.001	0.000	0.000	0.000	0.000	0.000	0.000	0.000	0.000	0.000	0.000	0.000	0.000

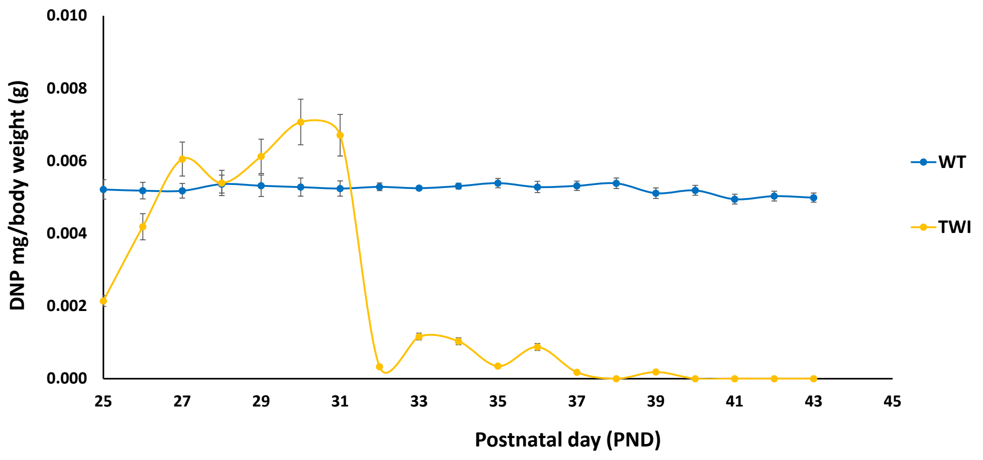

Figure 2. Mean daily DNP dose (mg)/per animal (± SD), for wild type (blue line) and twitcher mice (yellow line).
